# The Meaning of Signs: Diagnosing the French Pox in Early Modern Augsburg

**DOI:** 10.1353/bhm.2006.0142

**Published:** 2006

**Authors:** Claudia Stein

**Keywords:** French pox, Augsburg, medical semiotics, diagnosis, hospital

## Abstract

This article reconstructs the diagnostic act of the French pox in the French-disease hospital of sixteenth-century Augsburg. It focuses on how the participants in the clinical encounter imagined the configuration of the pox and its localization in the human body. Of central importance for answering this question is the early modern conception of physical signs. It has been argued that it was due to a specific understanding of bodily signs and their relationship to a disease and its causes, that disease definition and classification in the early modern period showed a high degree of flexibility and fluidity. This paper looks at how the sixteenth-century theoretical conception of physical signs not only shaped the diagnosis and treatment of the pox but also reflected the overall organization of institutions.

In December 1618 Philip Ess, a day laborer, presented himself at the municipal French-disease or pox hospital (*Blatterhaus*) in Augsburg.^1^The town council had founded the institution in 1495, the very year the pox made its first appearance in the city.^2^Since the mid-sixteenth century a [Other P-617] biconfessional hospital, the *Blatterhaus* was one of the many municipal and private health-care institutions aiming to bring relief to Augsburg's Protestant and Catholic poor. With a maximum capacity of sixty to seventy patients, it was an important pillar of the city's poor-relief system.^3^

Interestingly, during the course of the sixteenth century the impecuniousness of applicants became decreasingly important to the *Blatterhaus*'s administrators; instead, starting in the 1530s, it was the sufferer's physical condition that determined admission. This trend, detectable in hospital records and admissions rules, is very similar to what Colin Jones has argued for early modern French hospitals^4^—and in Augsburg, as in many French cities, it went hand in hand with the specialization of all of the city's healthcare institutions. By the mid-sixteenth century a physical examination (*Geschau*)—usually conducted by a barber-surgeon and a university-trained physician, and in the presence of the administrator, the caretaker, and other lay witnesses—decided whether the applicant's physical state met the specific requirements of the institution to which admission was sought.^5^

Philip Ess turned out to be a rather controversial case for the appointed medical experts of the pox hospital. After examining his naked body in detail, they agreed on the following diagnosis: his physical signs, they claimed, were ambiguous; they simultaneously pointed to the French disease and to an ailment that they identified as “elephantiasis.”^6^ (The latter was classified as one of the four possible forms of leprosy in the popular sixteenth-century vernacular medical treatise written by the Augsburg pharmacist Christoph Wirsung).^7^ Because Ess's physical signs indicating the French disease outnumbered those pointing to elephantiasis, the two practitioners concluded that a cure at their institution would probably be beneficial. Since he fulfilled all the other requirements for admission—citizenship, poverty, and evidence of an irreproachable family and community life—the hospital's civic administrators followed the practitioners' recommendation and admitted him to the male ward.

But only a few weeks later Ess's wretched body reappeared in the hospital records. His bodily signs had reattracted the attention of the hospital's medical practitioners on one of their daily rounds. After keeping him under close supervision for a few days, the physician and the barber-surgeon came to the conclusion that his physical signs had transformed into unambiguous signs of leprosy. This new diagnosis triggered instant and dire consequences for Ess's institutional fate: he was now considered a serious health threat to his fellow convalescent inmates and was immediately expelled from the *Blatterhaus*. The sources unfortunately do not tell us whether he applied for admission at one of the city's three leprosy hospitals (S. Servatius, S. Sebastian, and S. Wolfgang); from similar cases, however, we know that this would have been a likely option.^8^

Philip Ess's diagnosis is not the only one of its kind to be found in the surviving records. Within days a disease could metamorphose into another—or, as the Ess case shows, physical signs could reveal that a patient's body harbored several diseases simultaneously. What do these cases tell us about the identifcation and classifcation of diseases in the Augsburg pox hospital, and in the early modern period in general?[Other P-619]

I am not the first historian to ask such questions. The changing process and the varying methods of physical diagnosis in the past have long been among the most challenging topics in the history of medicine.^9^ In a recent article Andrew Cunningham claims that neither the outdated strategy of retrospective diagnosis nor the more recent study of changing disease concepts actually brings us closer to the identity of diseases in the past.^10^ Unlike retrospective diagnosis, the notion of disease concepts does take into account that diseases are not stable, ahistorical “scientifc facts” but changing sociocultural constructions. However, there lurks the danger of treating past disease concepts merely as the result of “things thought,” or as the more-or-less exclusive result of mental activity.^11^ Instead, Cunningham suggests, we should turn our attention toward *disease concepts in action*—that is, the diagnostic act itself: it is through the questions that are asked and answered during the act of diagnosis, he suggests, *and* through the practices applied by those involved in this operation, that the identity of disease (past and present) may be established.^12^

In this paper I take up Cunningham's suggestion. Through the investigation of a unique collection of archival hospital records, I shall reconstruct the diagnostic act in order to investigate the pox's identity within a specific institutional and local setting: the sixteenth- and early seventeenth-century municipal French-disease hospital in Augsburg. Too often the question of pox identity is swept under the carpet in the belief that the early modern French disease was in fact our modern disease entity venereal syphilis.^13^ Archival accounts, such as the case of Philip Ess, render this identifcation both difficult and questionable.[Other P-620]

In recent studies Ian Maclean and Nancy Siraisi have explored philosophical discussions on disease identification in early modern treatises on semiology.^14^ Their erudite and thorough analyses are extremely helpful in understanding early modern conceptions of disease definition and distinction. They show that these were ultimately based on a completely different understanding from today of the meaning of sensible bodily signs and their relation to disease and its causes. Both authors stress the multiplicity and complexity of the medical phenomena that early modern medical diagnosis had to reckon with, and that, as Siraisi points out, accounted for “the flexibility of definition and classification possible even in the case of well known and relatively well understood ailments.”^15^

But to what extent did these highly abstract theoretical discussions (mostly between university physicians trained in scholastic logic and rhetoric) shape the diagnostic practice at Augsburg's French-disease hospital? From the archival record we know that the busy daily routine of the house left very little time for sophisticated philosophical debate. Following Siraisi on the flexibility of disease definition and classification in the early modern period, I wish to explore some of the factors that might have guided the diagnostic decisions made about the meaning of physical signs in particular cases. What were the styles of reasoning and the various practices operating in the decipherment of physical signs at the *Blatterhaus* that ultimately allowed for the fluidity of disease definition and classification?^16^

My investigation is based on the assumption that although the participants in the examination differed widely in their social status and vocational background, they shared similar views and practices about the functioning of the human body, its diseases, and its treatment. Patients, practitioners, and witnesses in Augsburg's pox hospital, I maintain, all spoke essentially the same language; they were equal partners in a “unitarian medical world.”^17^ From archival records it is clear that Augsburg's sick[Other P-621] from all social and intellectual levels not only diagnosed their individual bodily signs and symptoms but also considered themselves competent judges of medical outcomes.^18^ As we shall see, the mind and the body of an ailing individual who presented him- or herself at the French-disease hospital was not a blank tablet on which the institution's medical practitioners simply stamped their conceptions of disease causation and localization. On the contrary, the mind of the sick person was a dynamic space for interior visualization and storytelling, which every healer, whether university-trained or not, had to take into account. The taking of a patient's history, the evaluation of his or her outwardly visible signs, and a thorough physical examination were integral parts (albeit often intertwined) of the clinical encounter between the medical practitioners at the Augsburg pox hospital and its patients. The diagnostic result of these individual subtle evaluations—or “medical portraits,” as they have recently been labeled^19^—was a merger between the medical practitioner's “sketches” of the patient's condition and the patient's (and, in the case of the French-disease hospital, the other witnesses') previous and/or simultaneous engagement in the same matter. The final diagnosis, I argue, presented a picture of disease that seemed to all the parties involved a meaningful and explanatory reflection of the patient's condition.

But how can we revive elements of Augsburg's unitarian medical world? In addition to the surviving hospital records revealing the daily practice at the pox hospital, I shall draw here on several vernacular German books on medicine and treatises on the pox. I am aware that this approach is methodologically problematic, not least because we can never be sure that any of the parties involved knew the contents of these books or were aware of their existence, even though some of them were written by Augsburg citizens.

Nevertheless, as Martin Giesecke has recently made clear for early modern Germany, vernacular books can teach us something about shared medical worlds.^20^ Very much like the “medical portraiture” emerging from [Other P-622] the clinical encounter, vernacular prints are products of collective consent. German medical texts, predominantly penned by those involved in the medical trade but with differing intellectual and social backgrounds, were all aimed at instructing the “common man”—that stratum of early modern society who did not read Latin easily or at all.^21^ To reach and instruct a wider, nonacademic, public a vernacular author had to anticipate, take into account, and build upon a potential buyer's, reader's, or listener's own knowledge and practical skills in that particular area. As Adrian Johns has claimed, early modern audiences for printed materials on the natural world, including the human body, were ( just like patients, I would add) not passive receptacles of information but generally positive agents of appropriation who had their own means of determining the credible and trustworthy.^22^ In order to become a “best-seller”—and this consideration played an increasing role in the minds of both author and publisher—a vernacular medical book needed to have the right mixture of “instruction” (describing something new) and “recognition” (building on the existing knowledge of the reader or listener). Vernacular German authors, Giesecke has argued, although they shared with Latin authors the same stock of knowledge about the natural world and the human body and accepted the same underlying logic, achieved the correct balance by downplaying complicated scholastic methodology while emphasizing practical applications.^23^ In vernacular printed material, the scholastic principle of argumentation and proof (“why”) takes second place behind description of knowledge and its application (“how”). In my concern with both the practical dimension of diagnosis and the underlying thinking that instructed that act, vernacular medical treatises are therefore a helpful source. But let us return to the examination room of the French-disease hospital.[Other P-623]

## The Examination

What actually happened during the examination of Philip Ess? From various house regulations and employment contracts, we know that the university-trained physician and the barber-surgeon were expected to perform different, if complementary, tasks. Ess's visible outer body was the exclusive domain of the barber-surgeon, who examined lesions and ulcers, and cleaned and dressed them; he also drew blood, applied leeches, and administered enemas.^24^The university-trained physician, on the other hand, focused on the inner body. Hospital employment contracts reveal that, with the help of his knowledge of ancient medical authors and Aristotelian natural philosophy, he was to decipher the hidden, invisible “universalia” of the sick body.^25^ They also show that his academic knowledge and degree made him a dispenser of directions not only to the sick but also to all nonacademic practitioners. In 1562, for example, the barber-surgeon Hans Gablinger was not allowed to perform any of his services—ranging from cleaning and dressing wounds, purging, and bloodletting to the administration of the dangerous mercury ointment or amputations of various body parts—without the explicit consent and under the close supervision of the physician, Daniel Zeller. In fact, it was not only the barber-surgeon whose work was supervised by the much-better-paid physician: the entire daily organization of the French-disease hospital was shaped according to the recommendations and will of the appointed learned physician.^26^

Why did the examination of the French disease in Augsburg's hospital involve two different kinds of practitioners with different responsibilities and authorities over the human body? Why was their close cooperation,[Other P-624] their “teamwork,” obligatory and considered obviously necessary for both the diagnosis and the treatment of the French disease?

## Seeing and Knowing

Today, as in early modern Augsburg, the core of medical diagnosis consists of the collection and interpretation of the patient's sensible physical signs. I shall argue that the hierarchy of healers in the *Blatterhaus* points to a specific understanding of physical signs and their relationship to disease. Although Philip Ess's bodily symptoms were taken care of by the barber-surgeon, they were not considered to be the disease itself. The higher salary and greater responsibility of the academic physician suggest that it was the investigation of the invisible realm of physical signs that was considered crucial for the understanding of their relationship to disease. Physical signs certainly indicate illness, but only insofar as they “allow the invariable form of the disease—set back somewhat, visible and invisible—to *show through*,”as Michel Foucault once put it.^27^ The invisible was a powerful organizational force; in the Augsburg pox hospital, it structured not only the healers' authority over the human body and the professional hierarchy, but also the entire institutional space.

To value some hidden essence of a bodily sign over its visible perception presents difficulties regarding the question of the identity of disease, at least for our modern understanding. Today, physical signs generally serve to indicate a specific nosological entity.^28^ When we talk about disease we have in mind some particular conditions.^29^ The term “disease” commonly refers to a pattern of signs that somehow hang together and recur, more or less the same, in successive individuals. It is only the recurrence of a pattern of events, a number of elements combined in a definite relationship,[Other P-625] chronological and geographical, that we label a disease. A disease consists of a congeries of signs—no single sign, by itself, makes a disease entity.

On the basis of this understanding, modern medicine tends to differentiate between subjective “symptoms,” felt only by the sick person, and objective “signs” that can also be observed by another person.^30^ That other person is usually the physician, who is expected to organize the “chaos” of subjective symptoms and to arrange them into a logical, coherent order, associated with a specific disease entity. It appears that there is no hidden meaning beyond the visible sign or symptom, which seems entirely transparent to the calculating and authoritative gaze and the language of the doctor.^31^

However, our understanding of the relationship between a bodily sign and disease would have struck the historical actors involved in the patient examinations at the Augsburg pox hospital as bizarre.^32^ The way they perceived and interpreted the physical reality of the patient's body permitted no space for our radical distinction between objective bodily knowledge “owned” by the medical practitioner and (in regard to its “truth” value) the subordinated subjective bodily knowledge felt by a patient. At that [Other P-626] time, a disease and its signs were bound together by a different code; crucially, they followed a different structure of visibility.

## Perceiving and Assigning Bodily Signs

At the base of the early modern structure of visibility stood a very different conception of physical signs. In his best-selling vernacular treatise *Spiegel der Artzney* (*Mirror of Medicine*), the Colmar physician Lorenz Fries submited that “a sign is a thing that explains something through its sensuality.”^33^ In other words, through its physicality a sign hints at something hidden from the beholder. Fries seems to say that the sensual experience of a physical sign alone holds no definite meaning in itself. His definition points to some kind of meaning-giving domain beyond the actual sensible. Recent work on the sense of sight and embodiment in the Middle Ages and the early modern period helps us to get closer to a different understanding of visibility and, hence, to what Fries may have had in mind.^34^ Medieval visual culture, it has been argued, can be grasped only if we allow ourselves to embrace the idea that in the past the visual was not solely visible: we have to account for an “invisibility of vision.”^35^ For medieval and early modern contemporaries, vision extended beyond mere sense perception and included extrasensory realms (terrestrial and spiritual conceptions and ideologies) of the world. From a modern perspective what is missing from this particular understanding of the visible is the perceptual correlate of an objective world—that is, the distinction between an objective and a subjective gaze (the objectifying gaze) that stands at the core of our modern conception of scientifc medicine and its perception of bodily signs.

Fries's definition of a physical sign was couched in Aristotelian philosophy.^36^ Indeed, he explicitly acknowledges Aristotle at the beginning of his[Other P-627] chapter entitled “Signs of Disease” as the great Greek philosopher who maintained that “all art should start from the things which are the most sensible to the investigator.”^37^ Fries's advice was related to the Aristotelian idea that the process of acquiring knowledge of any natural object began with the senses. From repeated sense experience followed memory; and from memory, by process of intuition, the experienced investigator of nature (in our case, the medical practitioner) was supposed to be able to grasp the universal feature of things, their nature or essence (the universalia). Once he possessed this universal definition, he could put it to use as the premise for deductive demonstrations. Thus, certain knowledge about the French pox was (for the academic physician at least) to be gained by a process that began with sense experience. However, what was learned by this inductive process did not acquire the status of certain and universal knowledge until it was put into deductive form. The end product of this process, then, was a deductive demonstration based on the universal definition of the natural object, such as the essence or nature of the French disease. But knowledge of the universal nature of the pox was not only necessary for philosophical deductive demonstrations: as we shall see, it was also the precondition of all treatment in Augsburg's pox hospital. In order to cure the pox successfully and without harm to the patient, knowledge of its invisible essence was thought absolutely vital.

But before I turn to that, let me investigate how early modern contemporaries imagined these universal features, the essence or nature of natural things such as the pox. “Nature,” Lorenz Fries explained, “is nothing less than an implanted force in all things that dwell under the moon. This force drives a thing to do things in the way it does them, or inspires it to strive for it.”^38^ Again, Fries's source for his short definition is the “prince” of all philosophers, Aristotle. Each natural object in the terrestrial sphere of the Aristotelian earth-centered and twofold cosmos was endowed with an individual inner force;^39^ this nature or essence was [Other P-628] unique to that object and strove incessantly to provide or to reinforce that specifc character, the particular behavior of the thing. In sixteenth-century Christian Western society, this force was believed to have been implanted by God in the act of divine creation.^40^

Diagnosis and treatment at Augsburg's French-disease hospital was firmly based on this understanding of each human body guided by its very own individual nature. This unique inner force acted in the body but could not be located. Nor was it permanently settled, in any organ or body part. The highest intention of human nature was to protect the body from any harm. For this purpose it was endowed with its own instrument for removing possible damage: excretion, the expulsion of disrupted disease-matter. In the case of diseases like the pox, sixteenth-century Germans such as the famous knight and humanist Ulrich von Hutten imagined the inner force of the human body as engaged in a fierce battle with the essence of the disease. Von Hutten had firsthand experience with the pox: his body had been ravaged by it for more than nine years, until in 1519 he was miraculously cured in Augsburg by the new “wonder drug” guaiacum (a wood imported from the newly discovered West Indies).^41^ In a tract published after his recovery, he described how the nature of the French disease tried incessantly to overpower the nature of his body, which strove (sometimes with the help of medication) to expel the disease's poisonous material through its various orifices (skin pores, mouth, ears, nose, anus, etc.).^42^ For von Hutten, as for most of his contemporaries, external bodily signs such as the many lesions and ulcers that covered his body [Other P-629] were not merely indicators of the invisible struggle going on inside the body between the nature of his body and its attacking disease(s); rather, they were accepted as proof that the body's nature had not yet submitted to the disease's sly onslaught.

It is, however, crucial to understand that the notion of the essence or nature of a disease such as the pox is a metaphysical concept. It is not to be confused with today's idea of disease entities, in which a set of signs is often regarded as firmly attached to a specific illness.^43^ For many of us today, signs and disease possess a causal and temporal sequence: a cause produces a disease, which in turn produces physical symptoms. For those who examined Philip Ess, however, nothing definite was understood about the relationship of a sign to a disease, its causes and its essence. While signs might have indicated the location of the disease inside the body, they did not necessarily reveal any of its essence.

This notion of a physical sign was supported and reinforced by the early modern idea of a dualistic body, a major symbolic opposition in Western medicine from its first formulation in the ancient Greek Hippocratic treatises.^44^ As I have indicated, in the case of Augsburg's *Blatterhaus* this ancient distinction between an inner and an outer body not only anchored the professional relationship between the two appointed healers, it also shaped and guided all diagnosis and treatment. It was agreed that the patient's inner body was a place of hidden activities; only through the physical sign on the surface of the skin could a patient and his or her healers speculate about the secrets inside.

This “unstructured osmotic space”^45^ of the patient's inner body was thought to be controlled by the incessant movement, the mixing and clashing, of its bodily fluids—the four humors—and their corresponding qualities (hot, cold, wet, and dry). In Hippocratic-Galenic medicine, which reigned paramount in the pox hospital until far into the seventeenth century, the four humors—blood (hot and wet), phlegm (cold and wet), black bile (cold and dry), and yellow bile (hot and dry)—were the body's primary elements, and were seen as more important than its solid organs: they regulated all the body's physiological functions and were believed [Other P-630] to be fused in the blood (the actual fluid found in the veins) and to be “concocted” from food in the liver.^46^

The hospital records concerning the treatment of the French disease show that the pox was conceived as deriving from a patient's particular complexion or temperament—that is, his or her individual and natural mixture of humors.^47^ The complexion not only disposed a person toward certain physical and mental characteristics, but also explained that person's susceptibility to certain diseases; it could become “unnatural” either by corruption or by an excess or deficiency of one or more humors and their qualities. While the hospital's medical practitioners viewed the blood in the body's veins in which the four humors were fused as the main seat of illness, they considered the humors diffused in the nutritional blood as the fountainhead of corruption and rotten matter associated with disease.

Again, one has to be careful not to fall back into modern modes of interpretation. The corrupted matter, ejected from the human body by its own nature, indicated to both the patient and the medical practitioner that something was occurring in the body. It could point to the essence of the disease, but on no account was it the essence itself.^48^ Unfortunately, the archival documents do not give us a concrete and definite answer to what the hospital's medical practitioners believed to be the essence of the pox. We do know, however, that when the disease first appeared in the last decade of the sixteenth century, Augsburg's academic doctors were engaged in a fierce controversy over it—though an agreement was soon reached.^49^ The hospital records indicate that from the early 1520s, at the latest, all treatment was based on the knowledge of the pox's essence. It is from the treatment of the pox that we can reconstruct at least some of the qualities that were associated with its alleged essence.

According to the general therapeutic guidelines of the predominant Hippocratic-Galenic medicine^50^ each disease was to be treated with[Other P-631] regimens and medications of opposite qualities to the ailment itself.^51^ Thus a disease of cold and moist quality, for example, would be best treated with a hot and dry regimen in order to bring the person's imbalanced complexion back to its assumed natural state. After 1522, all patients in the *Blatterhaus*, regardless of age, sex, or physical condition, were first treated with the wood guaiacum: administered as a hot drink and followed by an intense sweating cure. Thus it was thought to be very effective for the French disease but harmless to the overall condition of the patient's body.^52^ “The Indian wood is of warm nature and dry in the second degree. Therefore it is little wonder that it heals those sick persons and diseases which are of a cold and humid nature,” wrote one of Germany's most prolific sixteenth-century medical writers, Walter Ryff.^53^ The early seventeenth-century Augsburg barber-surgeon Joseph Schmid, who had firsthand experience through his own work in the city's pox hospital, held a similar vision: guaiacum warms and dries out the bodily humors, making them thin and fluid, he explained, so that they can then be easily expelled from the body.^54^

The mild guaiacum cure was not always sufficient, and severer cases of the pox in the *Blatterhaus* were subjected to mercury ointments or inhalations. These were dreaded for their toxicity by patients and medical practitioners [Other P-632] alike, with good reason.^55^ Ulrich von Hutten's account of some of the terrible effects of his eleven mercury cures (!), including intensive sweating, incessant salivation, and constant diarrhea, along with the loss of all body hair and teeth, still sends shivers down the modern reader's spine.^56^ Patients nevertheless endured these horrific effects because the incessant expulsion of all kinds of bodily fluids suggested to them that the pox was losing ground in its battle against the nature of their bodies and might finally be ready to leave them. Mercury was a treatment of last resort, however, as the rules of the *Blatterhaus* made clear: medical practitioners were advised to use this dangerous “acid cure” treatment only with moderation, and only after every other remedy had been tried.^57^

Since antiquity it had been known that the use of mercury necessitated the utmost caution.^58^ In Augsburg, patients treated with mercury were kept in a separate room so that other patients were not subjected to the dangerous smoke or the gruesome sight of the cure's dreadful accompanying symptoms. The Augsburg surgeon Joseph Schmid advised those of his colleagues dealing with mercury either to make the patient anoint him- or herself or, if the patient was too weak, always to wear protective gloves while administering it.^59^ The Nuremberg barber-surgeon Franz Renner suffered from “mercury phobia,” and reflected that for the whole of his career he had never applied mercury with his own hands; it was well known, he reminded his readers, that mercury “is damaging for the practitioner but a medication for the patient.”^60^ But, although the use of guaiacum and mercury was accompanied by different degrees of hope and fear, they had something important in common: they were both believed to be of a warm or hot and dry quality, and hence were considered suitable for the treatment of pox, a disease of a rather cold, moist, and acid nature.[Other P-633]

Just as Hippocratic-Galenic medicine tended to stress the uniqueness of each patient—natural complexion as well as imbalance—so each patient suffered from an individual “version” of the qualitatively cold and moist French pox. The enormous variety of signs and symptoms was a topic much discussed in vernacular treatises on the French disease. The Ansbach physician Tobias Knobloch, for example, boasted that he had successfully detected and treated more than three hundred different species of the French disease during his medical career.^61^ The self-appointed pox specialist and surgeon Joseph Schmid claimed that the hunt for the pox and its physical signs in each patient's body required an extremely experienced healer (he was certainly describing himself), for the signs of the pox were innumerable and they easily “fooled” healer and patient alike.^62^

Despite these alleged difficulties, the authors of pox treatises provided their readers with a list of symptoms that they personally considered to be related to the French disease. Each of them stressed that his list presented only a selection based on his own experience with the ailment and must under no circumstances be generalized. To present a complete list of the possible signs of the more than three hundred species of the pox, Knobloch explained, would have made his treatise unattractively long. Moreover, it would have led to unnecessary confusion among the laity that he sought to reach.^63^ Two forms of physical signs dominate the long lists of possibly pox-related symptoms: those occurring visibly on the skin of the victim (lesions, ulcers, etc.), and severe pain in the bones and joints. According to humoral theory, the signs appearing on the skin of a patient were generally associated with four different forms or species of the pox: melancholic, phlegmatic, choleric, and sanguinic. Judging carefully from its shape, smell, consistency, and color, each sign could be assigned to one of the four species. According to Franz Renner, for example, the choleric type tended to be dry and hard, reddish at the bottom, with a hard and yellowish lid, often accompanied by poisonous, voracious rashes. But the most dangerous species of pox, in his opinion, was the phlegmatic one: It tended to spread all over the body, and was of a liquid and suppurate consistency. Every area of the body it “poured into,” Renner explained, became affected and the skin was destroyed. Often the lesions did not [Other P-634] appear alone but were accompanied by some sort of acid, liquid, and incurable “scabies.”^64^

As to the other form of physical signs—pain in the bones and joints— this was explained as resulting either from a rapid change of the body's material structure or from a transformation of its qualitative composition.^65^ The latter was the reason for extreme feelings of pain in the French disease, according to Lorenz Fries in his *Spiegel der Artzney*: it was the particularly cold, acid, and wet quality of the pox-matter that caused excruciating pain in most complexions because it was foreign to them.^66^

Pain resulting from the pox was associated with two characteristics. First, the victims often were under the impression that the pain incessantly moved all over the body. The physician Alexander Seitz, who wrote a pox treatise on demand for Elisabeth Schott, the abbess of a nunnery close to his hometown of Marbach, related this phenomenon to the idea to be found in Galen's writings according to which pain was some kind of material substance: “A back-and-forth moving pain,” he explained, “is nothing other than a vapor or wind, according to Galen.”^67^ This pain-vapor was rooted in the poisonous disease-matter itself. Sometimes, however, the matter would expel the vapor and would then “chase” it throughout the body's veins. Hence the feeling that patients had of pain moving incessantly all over their body. In some cases, the pain-vapor could get stuck in very small veins and would slowly turn “into a matter equal to that it originated from” this vapor-turned-disease-matter could cause great destruction to the materiality of the inner body and was, according to Seitz, one of the causes for the “inner pox” that he had witnessed during his anatomy lessons at the University of Padua.^68^

As an explanation for the second widespread pain characteristic, the increase of its intensity during the night, Renner's view was standard: During the day the body's pores were wide open, so that poisonous humors could exit; at night, however, the pores closed, imprisoning the bad and unclean vapors and making their effects the more strongly felt.^69^

The lists of symptoms related to the pox provided by authors like Renner, Seitz, and Knobloch reveal a particular understanding of physical signs. Through its own sensuality a sign always indicated a change in the body, but these authors seem not to have linked any sign automatically to a particular disease. Signs such as pain were granted some kind of independent status, as the late French medical historian Roselyne Rey explained. Pain in early modern medicine, she argued, was viewed neither as an alarm signal nor as a sentinel; for an early modern medical practitioner, it was neither a state that announced an illness to come nor even a prodrome: it could already be the disorder itself.^70^

The disappearance of pain was therefore not inevitably a sign of recovery from the pox. Fries, for example, argued that it could equally be a hint that the nature of the body had become accustomed to the pox-matter and had given up fighting against it; in this dangerous case, the disease-matter, the great trickster, could spread unnoticed throughout the body, and far worse things could be expected.^71^ By this reasoning, Fries strongly opposed any use of painkillers. For him as for all the other authors, the materiality of a bodily sign possessed solely an indicating character.

## The Pox and the Body

This peculiarity of signs, their general interpretive openness, accounted for a particular understanding of the relationship between the pox and the body in which it was lodged. From the archives and the pox treatises emerges a picture similar to what Foucault described for the preclinical period: “The nosological picture of the disease,” he argued, “involves a figure of the disease that is neither the chain of causes and effects nor the chronological series of events nor its visible trajectory in the human body.”^72^ The set of bodily signs that defined the relation of the French disease to the organism was believed to be neither constant nor necessary. The pox was not defined by a recurrent pattern of symptoms and [Other P-636] events, assembled in a specific chronological and geographical order. In the understanding of the patient and the medical practitioners in the French-disease hospital, the body and the pox did not possess a common, previously defined space; they communicated solely through the nonspatial element of the individual patient's humors and qualities.^73^ As noted above, the diagnostic and therapeutic gaze was directed upon that which was invisible in the patient's sick body, the very nature or essence of disease. It was this underlying metaphysical concept that accounted for a certain constancy and permanence of disease, even if the physical signs associated with it differed from case to case.^74^

The position of the disease in the body was therefore of minor importance. Although Hippocratic-Galenic medicine assumed some kind of relationship between bodily and functional damage, autopsy findings (and there is no evidence that postmortems were ever performed in Augsburg's pox hospital) were considered inconclusive regarding conditions in the undisturbed depths of the living body: the visible internal devastation of organs in a dead body would not be automatically understood as the disease's visible way through the living body.^75^ In fact, the pox circulated freely in corporeal space. Nobody knew this better than von Hutten: he related how the pox would appear suddenly and simultaneously in various locations of his body, in all kinds of forms. But even worse, the tormented knight complained, sometimes it chose a secret, protected hiding place; in this “castle” it would remain unnoticed, quietly mustering strength in order to attack and “pour itself” again into all areas of the body at some unexpected moment.^76^

The pox was not only a “homeless” ailment: it also escaped time. In sixteenth-century pathology, time did not play an important role.^77^ It was admitted that a disease may last, and that its various episodes may appear in turn; since antiquity medical practitioners have calculated the critical days of a disease, and have known the significant values of arterial pulsation.^78^ All authors of pox treatises thus separated different phases or stages [Other P-637] of the disease, each phase being characterized by a different quantity of assumed disease-matter.^79^ In the first stage, the amount of the poisonous pox-matter was thought to be still very small and thus rather powerless in comparison to the body's nature. Treatment during this early stage was considered relatively easy: the disease-matter could be successfully diminished and expelled from the body through sweating, purging, and phlebotomy, Joseph Schmid suggested.^80^ The second phase, in which the disease-matter had considerably increased, was characterized by a fiercer battle between the disease and the nature of the body. This invisible internal fight would manifest itself in a sharp increase in the number of outwardly sensible signs. The internal struggle culminated in the crisis of the pox, the moment when a sudden and dramatic change in the course of the disease (either toward recovery or, in the worst case, toward death) could be expected. Traditionally, the seventh, fourteenth, and twenty-first days after the beginning of the disease were considered especially important in this regard. A crisis could be expected, for example, when symptoms were no longer on the increase.

This numerable fixed duration of an illness was part of the essential structure of any disease, and the French pox did not in this respect differ from other ailments such as leprosy or dropsy. What did not exist in the minds of the sick and the medical practitioners at the Augsburg French-disease hospital was the idea that a disease evolved, or that it spontaneously introduced new events of itself. Again, as Foucault perceived, “time is integrated as a nosological constant, not as an organic variable. The time of the body does not affect, and still less determines, the time of the disease.”^81^

Neither the medical practitioner in the French-disease hospital nor any authors of the pox treatises could or would give patients and readers any idea as to the exact duration of each phase of their individual illness, or predict how long the pox would endure. Everything depended on the quantity of the disease-matter accumulated in the patient's body, and on his or her individual complexion and living conditions. However, some [Other P-638] writers, such as Alexander Seitz, did submit cautious generalizations based on their own practical experience and their knowledge of ancient medical literature.^82^ Patients with a rather wet and cold temperament (and this was especially the case with women), Seitz explained to the abbess Elisabeth, were less prone to attract the pox and could expect their suffering to be less severe.^83^ The nature of the bodies of these complexions, he continued, did not consider the cold and wet disease-matter as much different from its own natural qualities and consequently would not engage in a major fight against it. Patients with warmer and dryer complexions (such as men), however, could expect to be struck much harder. Their nature, in its qualities very much opposed to that of the pox, “disliked” the latter so intensely that it would undertake the most dramatic actions in order to get rid of it. This is how von Hutten understood his extraordinarily long and intense suffering: his warm and dry complexion, he had been told by a physician, in combination with his life style as a scholar, made him the perfect victim for the pox. Was it not well known, von Hutten asked his readers, that “in those who are raised delicately and are drawn to books, diseases heal much slower than in others?”^84^

No definite answers in relation to the duration of the pox could be given. As the Ansbach physician Knobloch bluntly informed his readers, “No time can be set.”^85^ And the reply of the Magdeburg physician Magnus Hundt to this question was widely shared even among authors from the later part of the sixteenth century and the early seventeenth century: “According to the complexion of the body, and the quality and quantity of the poisonous matter … some get rid of the disease soon, some slowly, and many drag themselves to the grave.”^86^

## What Was the French Disease?

I have argued that physical signs were not automatically assigned to the French disease. What, then, turned a physical sign into a sign of the pox in the French-disease hospital? It was only during the clinical encounter, as I aim to show in the following, that the significance of each physical sign was established for each individual case.

From the treatises cited above it becomes clear that during the act of diagnosis every tiny element of the patient's present and past life and living conditions had to be taken into account, and each was interrogated for its possible relation to the patient's suffering. The search for the meaning of bodily signs beyond their visible perception was embedded in the belief in an intimate micro-macrocosm association. This ancient worldview, according to which the human body, the microcosm, was an exact copy of the surrounding God-created macrocosm, played a fundamental role in the field of early modern knowledge about the human body. To define the French disease was to bring to light this system of resemblances and analogies that made the body and the surrounding macrocosm closer to and dependent upon one another.^87^

Thus, the all-decisive moment in which a physical sign on an applicant's body in the Augsburg French-disease hospital would turn into a sign or symptom of the French disease was the encounter of the sufferer with the two healers in the institution's consulting room. This was the moment when the sufferer's personal disease-narrative became of the utmost importance. Part of this story was already known to the two healers before they first met the patient, for everyone wishing to be considered for admission had to hand in an official application letter to the administrators several days prior to the actual examination. In the letter the sufferer explained his or her pecuniary situation and wider social circumstances; often a short description of symptoms was added, and some proposed reasons for the cause of their illness. The soldier Hans Mayr, for example, related his illness to the cold and wet weather conditions encountered on a recent long ship journey.^88^ The wet nurse Ursula Wagner, mother of [Other P-640] four small children, wondered in November 1600 whether she might have attracted the pox from one of the three other children she had recently wet-nursed: one of these children, she claimed, had died from what was diagnosed as the pox soon after she had started nursing it.^89^ An elderly day laborer, Jacob Koler, reasoned in March 1583 that he had most likely contracted the pox during a visit to one of the city's public baths the year before.^90^ A weaver, Lienhart Doerr, whose wife and four children suffered and eventually died from the pox, did not consider any natural cause sufficient to explain the extraordinarily horrible fate of his family in 1583: for him, the only valid explanation was the wrath of God, who had punished him and his loved-ones in order to set an example for all of mankind's sins.^91^

The hospital records do not reveal the actual conversations between medical practitioners and applicants at the moment of examination. However, Fries in *Spiegel der Artzney* points to areas where the conversation might have gone. Indeed, he provides his readers with a long list of daily habits that they should mention during the consultation with medical practitioners, in order to provide the basis for the best diagnosis possible. His list was loosely based on the so-called ancient six non-naturals, those influences to which the human body was incessantly exposed, such as air, sleep and waking, repletion and filling, and emotions. Any alteration of one's daily habits could trigger a substantial change in one's natural complexion, and might thus be responsible for causing the pox. Therefore the patient should recollect

whether the disease had appeared first on the fields or in the house, whether you felt cold or too hot, whether you were dressed or naked, how you dealt with drink and food—whether you have eaten at favorable times or not, or whether you had fasted too long or eaten too much, whether you have prepared the food wrongly or eaten an inappropriate foodstuff, or whether it was prepared inappropriately—whether you have been awake a lot or have slept. Also whether you were lazy or worked, or whether you are used to being lazy and whether you worked the moment you fell ill. Whether you have been angry, melancholic, frightened.^92^

These bits of information were indispensable for hospital practitioners in determining the meaning of signs as well as the individual's overall state of health. The latter was not assessed solely on clinical grounds [Other P-641] but also on financial considerations. The rules of the *Blatterhaus*, as well as the employment contracts of its medical practitioners, stressed that only those applicants with the French disease who had, according to the judgment of the practitioners, a fair chance of recovery were admissible. Hopeless cases and those who might be unable to survive the cure were to be turned down. The town council feared that otherwise, “much cost and medicine, effort and work [would be] wasted.”^93^

In the act of linking an applicant's personal disease-narrative to physical signs, both the medical practitioner and the barber-surgeon were guided by their intellectual knowledge about the configuration of the pox and its location in the body, as well as their long practical experience in treating it together. The records indicate that they usually worked along side each other for several years, sometimes even decades.^94^ Together they examined hundreds of bodies, and restored to health the great majority of their French-diseased patients. Thus, not only did they come to an agreement on the invisible essence of the pox, but they also reached a shared understanding of which signs in each patient pointed to the disease essence. Through mutually gained experience they were able to distinguish these signs from physical manifestations that were only accidental and fortuitous, dependent on a patient's individual nature, complexion, dwelling place, profession, age, astrological inclination, and so on.

It has been recently pointed out that the early modern art of diagnosis left a great deal to the judgment of the medical practitioners, and that it thus had much in common with some practices in Chinese medicine.^95^ Shigehisa Kuriyama has argued that the art of medical diagnosis in ancient and contemporary Chinese medicine grows out of a long perceptual education, akin to other arts such as wine-tasting or music appreciation.^96^ The barber-surgeon and the university-trained physician had no absolute scale by which to judge the qualities of their patients' temperaments; as has [Other P-642] been recently explained, “no machine or instrument existed to produce an objective measurement of the softness or hardness of the skin, and thus wetness or dryness of the underlying material.”^97^ The identification of signs related to the pox required from both practitioners at the *Blatterhaus* a talent for providing a viable interpretation that did not confict either with the principles of their individual intellectual education or with the experience they had gained in their daily medical practice.

## Metamorphosis and Identity

The absence of any standardized measurement in early modern medicine and the fact that invisible realities had to be included in the interpretation of sensible signs, which held no definite meaning in their relation to disease, help to explain the diagnosis of Philip Ess.

The pox in early modern Augsburg was perceived in a space of projection without depth, and of coincidence without development. It was a timeless and homeless disease, circulating in the body without owning a specific set of symptoms. Unlike diseases today, the pox was not understood as etiologically, morphologically, or symptomatically distinguishable from other ailments. Nor was it imagined as an entity that evolved. For those present at Ess's examination in December 1618 it was perfectly conceivable that his body could harbor the pox *and* leprosy and exhibit signs of both diseases simultaneously.

The vicinity of the two diseases in Ess's body was not defined by measurable distances, but rather by visible similarities and the many invisible analogies that linked the human body to the surrounding macrocosmic world. Von Hutten told his readers that the French disease and leprosy were such close “neighbors” and intimate “friends” that at some point they could merge into one another. This closeness could be explained, he argued, by the fact that they shared the same astrological ascendants.^98^ In the understanding of those present at Ess's examination, the pox and leprosy were separated only by the degree of their resemblance—and the degree to which they resembled each other was, as I have shown, largely a question of medical interpretation with the help of the patient. When in the healers' opinion the similarities and resemblances between two diseases became dense enough, they could cross the verge of mere kinship, friendship, or neighborhood and achieve unity of essence. In Ess's case, the pox metamorphosed into leprosy.[Other P-643]

But although each diagnosis left a great deal to the judgment of the two healers, the individual examined and the witnesses present had to agree with it. Some cases found in the hospital records show clearly that this did not always happen. The patients and their relatives had strong views on what physical signs meant, and they might insist on the accuracy of their own interpretation. A difficult case in this regard for the medical practitioners of the pox hospital was Walburga Reuchart, who brought her sick three-year-old daughter to be examined in May 1564.^99^ The girl was a serious and heartbreaking case: she was in terrible pain, her small body covered with open lesions and ulcers. However, Doctor Zeller and surgeon Hans Gablinger came to the conclusion that the girl's signs were not related to the French disease but rather to some kind of poisonous and infectious rash, which was not treated in the pox hospital. They therefore refused her admission and advised her to try the Holy Ghost hospital—an odd suggestion in view of the fact that this institution refused to accept children or any sufferers from diseases identifed as infectious.^100^

Walburga Reuchart was not satisfed with the practitioners' verdict. She identified her daughter's lesions as signs of the French disease. Although she had been widowed and was without any substantial financial means, she had managed to obtain the opinion of several healers in town, all of whom had confirmed her initial suspicion and, aware of her precarious financial situation, had advised her to apply for free treatment at the municipal pox hospital. While she was not convinced of Zeller's and Gablinger's diagnosis, and was perhaps unaware of the various health-care institutions' admissions practices, she initially followed their suggestion and took her daughter to the Holy Ghost hospital. In the official examination certificate, the hospital's three appointed physicians—Leopold Trenklin, Marcus Wind, and Bernhard Schludi—backed Walburga's interpretation.^101^ All three agreed that the girl's signs were unambiguously related to the French disease. According to the hospital's house rules, this diagnosis freed them from all responsibility for Walburga and her daughter.

The official diagnosis certificate, signed by three important members of Augsburg's medical elite, empowered Reuchart to return to the French-disease [Other P-644] hospital and officially question Zeller's and Gablinger's diagnosis. Both reacted furiously, not so much because of her reappearance (many sufferers showed up several times) but because of her audacity in openly defying their diagnosis with an official certificate issued by their own colleagues. They felt that their professional honor was being attacked. In a letter written to the town council, both bemoaned the three physicians' unsporting conduct. They also sought to control the smoldering reproach, sparked by Walburga and the physicians' certificate, that they had deliberately “misread” the girl's physical signs in order to avoid burdening themselves with the admission of a complicated and labor-intensive case of the pox. Zeller and Gablinger reemphasized their original verdict: in their professional opinion, based on their long experience with the pox, the little girl was not suffering from the French disease. Moreover, they added, even if they made a generous exception and admitted the girl, it was her overall physical state that stood against her admission: her body had been so badly ravished that little or no improvement could be expected. It was to be feared that she would become a *stetiger Krankher* (permanent patient) in the institution—and it was well known, they cleverly reminded the money-conscious hospital administrators (quoting the house rules), that they were not allowed to admit anyone whose recovery they had judged impossible.

## Conclusion

Recent scholarship has pointed to the great flexibility of early modern disease classification and definition. This paper is much indebted to these analyses. But my aim was to go beyond the mere textual and to include in the investigation the act of diagnosis itself. In locating the clinical encounter within the specific local and institutional setting of the municipal French-pox hospital in sixteenth-century Augsburg, I have sought to illustrate early modern medical semiotics *in action*. Thus, I have focused on how physical evidence (bodily symptoms and signs) was perceived and related to disease by those involved in the daily clinical encounter of the hospital. My investigation was based on the assumption that all participants in the encounter shared the same stock of knowledge about the functioning of the human body (microcosm) and its intimate relationship to the wider natural world (macrocosm), albeit to different degrees of rhetorical sophistication and personal practical experience. It was accepted that the search for the meaning of all immediate sensory perceptions had to be extended to a variety of extrasensory realms and had to take into account all manner of spiritual conceptions and ideologies as well as unlimited analogies[Other P-645] and interdependencies between the two cosmoses. Proper knowledge and diagnosis of the pox in Augsburg's French-disease hospital was gained not by the mere act of “seeing” the physical sign, but only by decoding its invisible meaning. Or, as Foucault has described it: “To know must therefore be to interpret: to find a way from the visible mark to that which is being said by it and which, without that mark, would lie like unspoken speech, dormant within things… . Divination is not a rival form of knowledge; it is part of the main body of knowledge itself.”^102^

The belief in the interpretation of the invisible to define disease shaped the way the diagnosis was conducted. At the same time, the power of the invisible also was reflected in the overall institutional organization of the pox hospital, particularly in the hierarchical order of the appointed medical practitioners. The Augsburg case study supports earlier suggestions put forward by studies on early modern London and Bologna, that the higher social and economic status of the academic physicians was to a large extent based on the widespread belief that their intimate knowledge of ancient medical authorities and Aristotelian natural philosophy was indispensable for the decipherment of the everlasting universalia of disease and human nature, hidden behind the mere sensible particulars. Only this knowledge, it was widely assumed, enabled a medical practitioner to steer a safe course through all the perils of illness. Thus, in the diagnosis of pox at the French-disease hospital in Augsburg, the physician's ability to investigate the realm of the invisible not only endowed his opinion with a somewhat higher degree of “truthfulness”, but also secured him a dominant position within the institutional hierarchy.

The unquestioned belief in the authority of the invisible also provided the basis for the equal partnership of patients, witnesses, and medical practitioners. In the absence of our modern distinction between a subjective and an objective gaze, as well as standardized procedures to measure bodily functions, the identity of the French disease, I contend, could be forged only in a process of discursive and nondiscursive mediations between the various parties involved in the very act of the diagnosis—the power of disease definition was equally shared among all participants. The opinion of academic physicians never carried absolute authority: it could, and often would, be contested by patients and their relatives (as shown by the case of Walburga Reuchart), or even by academic peers. Every diagnosis of the pox required the subtle art of evaluation and negotiation so that, in the final judgment, all parties involved accepted the medical portrait drawn for that particular case.[Other P-646]

The broader and more general question raised here is the extent to which the early modern understanding of disease was flexible, while coexisting with increasingly rigid institutional structures and admissions practices, tightened house rules, and more explicit professional hierarchies in the specialized health-care institutions of sixteenth-century Augsburg. However, it remains uncertain how far we can generalize from the evidence of one case study. Moreover, the “gaze” of the participants in the clinical encounter at the pox hospital may well have been constituted and guided by more than just their eagerness to identify the hidden universals beyond the immediate sensual particulars. If, as claimed above, proper knowledge of things (including the human body and its diseases) was to be gained only by interpretation, then economic motives and interests or political strategies and tactics, as well as personal animosities and rivalry among the participants in the clinical encounter, certainly must have played a significant role in their decision-making. If I have chosen not to emphasize and investigate these elements, it is only to concentrate more on the still neglected practical application of sixteenth-century medical semiotics.^103^ In particular, my concern has been to reconstruct from the archival sources how the participants in the clinical encounter at the Augsburg French-disease hospital imagined the configuration of the pox and its localization in the human body, and how this understanding was reflected in the treatment of the disease and the hierarchical order of the appointed medical practitioners.

## References

[b1-80.4stein] 1.For this case, see Suppliken an die Stadtpfeger, Bürgermeister und den Rat um die Aufnahme ins Blatterhaus 1548–1804, Stadtarchiv Augsburg, Reichsstadt, St. Martinsstiftung (hereafter SA), Karton VI, no. 47 (Prod. 1–121), Prod. 7a; Prod. 86.

[2] Claudia Stein, *Die Behandlung der Franzosenkrankheit in der Frühen Neuzeit am Beispiel Augsburgs* (Stuttgart: Steiner, 2003), pp. 115–39

[3] Stein, *Franzosenkrankheit* (n. 2), pp. 95–139

[4] Colin Jones, “The Construction of the Hospital Patient,” in Finzsch and Jütte, *Institutions and Confinement* (n. 2), pp. 55–74, on p. 60

[5] Stein, *Franzosenkrankheit* (n. 2), pp. 100–114, 118–19, 130–33, 139

[b6-80.4stein] 6.“Nit allein cum morbo gallico, sondern auch cum elephantiasis (SA, Karton VI, no. 47, Prod.86).”

[7] Christoph Wirsung, *Artzney Buch* (Heidelberg, 1586), pp. 510–12

[8] Stein, *Franzosenkrankheit* (n. 2), pp. 109–12

[9] Roy Porter, “The Rise of Physical Examination,” in *Medicine and the Five Senses*, ed. William Bynum and Roy Porter (Cambridge: Cambridge University Press, 1993), pp. 179–97

[10] Andrew Cunningham, “Identifying Diseases in the Past: Cutting through the Gordian Knot,” *Asclepio*, 2002, 54 (1): 13–34

[11] Cunningham, “Identifying Diseases” (n. 10), pp. 15–16

[12] Cunningham, possess an “operational identity”: “Identifying Diseases” (n. 10), p. 16

[13] Arrizabalaga, French and Henderson, *The Great Pox*(n. 2)

[14] Ian Maclean, *Logic, Signs and Nature in the Renaissance: The Case of Learned Medicine* (Cambridge: Cambridge University Press, 2002), pp. 282–84

[15] Siraisi, “Disease and Symptom” (n. 14), p. 239

[16] Ian Hacking, *Historical Ontology* (Cambridge: Harvard University Press, 2001), pp. 178–99

[17] Colin Jones and Laurence Brockliss, in *The Medical World of Early Modern France* (Cambridge: Cambridge University Press, 1997), p. 237,

[18] Gianna Pomata, *Contracting a Cure: Patients, Healers and the Law in Early Modern Bologna* (Baltimore: Johns Hopkins University Press, 1998), p. 123 15072043

[19] Brian Nance, *Turquet de Mayerne as Baroque Physician: The Art of Medical Portraiture* [Amsterdam: Rodopi, 2001], p. 78 11747519

[20] Michael Giesecke, *Der Buchdruck in der Frühen Neuzeit: Eine historische Fallstudie über die Durchsetzung neuer Informations- und Kommunikationstechniken* (Frankfurt: Suhrkamp, 1998), pp. 527–41

[21] Peter Blickle, *Deutsche Untertanen: Ein Widerspruch* (München: Beck, 1981), pp. 15–18

[22] Adrian Johns, *The Nature of the Book: Print and Knowledge in the Making* (Chicago: University of Chicago Press, 1998), pp. 31–43

[23] Giesecke, *Buchdruck* (n. 20), pp. 555–56

[24] Stein, *Franzosenkrankheit* (n. 2), pp. 155–62

[b25-80.4stein] 25.For the duties of learned physicians, see the contract of Doctor Daniel Zeller, 19 November 1562, SA, Karton VII, no. 50, Prod. 1.

[26] Stein, *Franzosenkrankheit* (n. 2), pp. 203–61

[27] Michel Foucault, *Birth of the Clinic: An Archaeology of Medical Perception* (New York: Vintage Books, 1994), p. 90

[28] Margaret Pelling, “Contagion/Germ Theory/Specificity,” in *Companion Encyclopaedia of the History of Medicine*, ed. W. F. Bynum and Roy Porter, vol. 1 (London: Routledge, 1993), pp. 309–34, on p. 314

[29] Lester King, “What Is Disease?” in *Concepts of Health and Disease: Interdisciplinary Perspective*, ed. Arthur Caplan et al. (Reading, Mass.: Addison-Wesley, 1981), pp. 107–18, on p. 112

[30] Wear, *Knowledge and Practice* (n. 20), p. 127

[31] *Birth of the Clinic* [n. 27], p. 91

[32] F. C. Crookshank, “The Importance of a Theory of Signs and a Critique of Language in the Study of Medicine,” in *The Meaning of Meaning: a Study of the Influence of Language upon Thought and the Science of Symolism, by C. K. Ogden and I. A. Richards with supplementary essays from B. Malinowsky and F. C. Crookshank*, 7th ed. (New York: Harcourt, Brace, 1945)

[33] Lorenz Fries, *Spiegel der Artzney* [Strasburg, 1532], fol. 39)

[34] Suzannah Biernoff, *Sight and Embodiment in the Middle Ages* (Basingstoke: Houndsmills, 2002)

[35] Biernoff, *Sight and Embodiment* (n. 34), p. 3

[36] G. E. R. Lloyd, *Aristotle: The Growth and Structure of His Thought* (Cambridge: Cambridge University Press, 1968)

[37] Fries, *Spiegel der Artzney*[n. 33], fol. 39)

[38] Fries,*Spiegel der Artzney*, fol. 3^r^)

[39] Robert S. Westman, “The Copernicans and the Churches,” in *God and Nature: Historical Essays on the Encounter between Christianity and Science*, ed. David C. Lindberg and Ronald L Numbers (Berkeley and Los Angeles: University of California Press, 1986), pp. 76–113

[40] David C. Lindberg, *The Beginnings of Western Science: The European Scientific Tradition in Philosophical, Religious, and Institutional Context, 600 B.C. to A.D. 1450* (Chicago: University of Chicago Press, 1992), p. 281

[41] Michael Peschke, *Ulrich von Hutten (1488–1523) als Kranker und als medizinischer Schriftsteller* (Cologne: Forschungsstelle des Instituts der Geschichte der Medizin in der Universität zu Köln, 1985)

[42] Ulrich von Hutten, *Ulrich vo[n] Hutten eins teutschen Ritters von der wunderbarliche[n] artzney des holtz Guaiacu[m] genant, und wie man die Frantzosen oder blattere[n] heilen sol … * (Augsburg, 1519)

[43] Nance, *Turquet de Mayerne* (n. 19), p. 115

[44] Pomata, *Contracting a Cure* (n. 18), p. 132

[45] Barbara Duden, *Women Beneath the Skin: A Doctor's Patients in Eighteenth-Century Germany* (Cambridge: Harvard University Press, 1991), p. 127

[46] Nancy Siraisi, *Medieval and Early Renaissance Medicine: An Introduction to Knowledge and Practice* (Chicago: University of Chicago Press, 1990), p. 106

[47] Stein, *Franzosenkrankheit* (n. 2), pp. 140–52, 203–39

[48] Nance, *Turquet de Mayerne* (n. 19), pp. 115–16

[49] Stein, *Franzosenkrankheit* (n. 2), pp. 140–52

[50] Joachim Telle, “Wolfgang Thalhauser: Zu Leben und Werk eines Augsburger Stadtarztes und seinen Beziehungen zu Paracelsus und Schwenkfeld,” *Medizinhistorisches Journal*, 1972, 7 : 1–30 11609764

[51] Siraisi, *Medieval and Early Renaissance Medicine* (n. 46), pp. 115–52

[52] Stein, *Franzosenkrankeit* (n. 2), pp. 143–52

[53] Walter Ryff, *New erfundne/ heylsame/ und bewärte artzney/ gewusse hilff unnd radt/ nit allein die Frantzosen oder bösen blatern/ … bissher für vnheylbar geacht worden/ gründlicher vnd gentzlicher zu vertreiben/ heylen vnd Curieren* (Strasburg, 1559)

[54] Joseph Schmid, *Kurtzer iedoch Gewisser bericht, dreyer Erblicher kranckheiten, alβ da sein, die Pest, Frantzosen, vnd Scharbock, wie sie mögen curirt werden* (Augsburg, 1667), pp. 173, 203

[55] Stein, *Franzosenkrankheit* (n. 2), pp. 209–12

[56] von Hutten, *Holtz Guaiacum* (n. 42), p. 409

[b57-80.4stein] 57.Blatterhaus, Ordnung von 1576, SA.

[58] Erna Lesky, “Von Schmier-und Räucherkuren zur modernen Syphilistherapie,” *Ciba-Zeitschrift*, 1959, 8 : 3174–89

[59] Schmid, *Kurtzer bericht* (n. 54), p. 202

[60] Franz Renner, *Ein new wol gegründet nützlichs vnnd haylsams handtbüchlein* (Nürnberg, 1559), fol. 30^r^

[61] Tobias Knobloch, *De Lue Venerea: Von den Frantzosen kurtzer Bericht* (Giessen, 1620), p. 32

[62] Schmid, *Kurtzer bericht* (n. 54), p. 146

[63] Knobloch, *De Lue Venerea* (n. 61), p. 32

[64] Renner, *Handtbüchlein*(n. 60), fols. 6^r^—7^r^

[65] Rosa María Moreno Rodríguez and Luis García-Ballester, “El dolor en la teoría y la práctica médicas de Galeno,” *Dynamis*, 1982, 2 : 3–24 11621614

[66] Fries, *Spiegel der Artzney*(n. 33), fol. 39^a^

[67] Alexander Seitz, *Ein nutzlich regiment wider die bösen frantzosen …* (Pforzheim, 1509)

[68] Alexander Seitz, *Ein nutzlich regiment wider die bösen frantzosen …* (Pforzheim, 1509)

[69] Renner, *Handtbüchlein*(n. 60), fol. 5^r-v^

[70] Rey, *History of Pain* (n. 65), p. 27

[71] Fries, *Spiegel der Artzney*(n. 33), fol. 33^v^

[72] Foucault, *Birth of the Clinic* (n. 27), p. 4

[73] Foucault, *Birth of the Clinic* (n. 27), p. 13

[74] Nance, *Turquet de Mayerne* (n. 19), pp. 114–15

[75] Jerome Bylebyl, “The Manifest and the Hidden in the Renaissance Clinic,” in Bynum and Porter, *Medicine and the Five Senses* (n. 9), pp. 40–60

[76] Von Hutten, *Holtz Guaicum* (n. 42), p. 306

[77] Foucault, *Birth of the Clinic* (n. 27), p. 12

[78] Ingo Wilhelm Müller, *Humoralpathologie: Physiologische, pathologische und therapeutische Grundlagen der galenischen Medizin* (Heidelberg: Haug, 1993), p. 108

[79] Stein, *Franzosenkrankheit* (n. 2), pp. 79–80

[80] Schmid, *Kurtzer bericht* (n. 54), p. 144

[81] Foucault, *Birth of the Clinic* (n. 27), p. 12

[82] Seitz, *Ein nutzlich regiment* (n. 67), p. 11

[83] Joan Cadden, *Meanings of Sex Difference in the Middle Ages: Medicine, Science, and Culture* (Cambridge: Cambridge University Press, 1993).

[84] Von Hutten, *Holtz Guaiacum* (n. 42), p. 476

[85] Knobloch, *De Lue Venerea* (n. 61), p. 123

[86] Magnus Hundt, *Eyn kurtzes vnd sehr nutzbarlichs Regiment wider dye schwynde vnd erschreckliche kranckheit der Pestilentz … Meher eyn nutzlichs Regiment wider die weltleuftige un[d] vnsauber kranckheit der Frantzosen* (Magdeburg, 1529), fol. 19^v^

[87] Stein, *Franzosenkrankheit*(n. 2), pp. 39–52

[b88-80.4stein] 88.SA, Karton VI, no. 47, Prod. 67.

[b89-80.4stein] 89.Ibid., Prod. 23.

[b90-80.4stein] 90.Ibid., Prod. 44.

[b91-80.4stein] 91.Ibid., Prod. 43.

[92] Fries, *Spiegel der Artzney*(n. 33), fol. 4^r^

[b93-80.4stein] 93.“Dieweil vil unbeschichketer schaden sein, darbey sy gehailt vnd gesund gemacht werden kain hoffnung ist … darbey zu besorgen, das aller uncost vnnd arztney mue vnd arbeitt verloren sey” (Blatterhaus, Ordnung von 1522, SA).

[94] Stein, *Franzosenkrankheit*(n. 2), pp. 238–39

[95] Nance, *Turquet de Mayerne*(n. 19), p. 98

[96] Shigehisa Kuriyama, “Pulse Diagnostic in the Greek and Chinese Traditions,” in *History of Diagnostics: Proceedings of the 9th International Symposium on the Comparative History of Medicine, East and West*, ed. Y. Kawakita (Osaka: Tangiguchio Foundation, 1987), pp. 43–67

[97] Nance, *Turquet de Mayerne* (n. 19), p. 98

[98] Von Hutten, *Holtz Guaicum* (n. 42), p. 406

[b99-80.4stein] 99.For this case see SA, Karton VI, no. 47, Prod. 35.

[100] Stein, *Franzosenkrankheit*(n. 2), pp. 105–7

[101] Elisabeth Martz, *Gesundheitswesen und Ärzte in Augsburg im 16. Jahrhundert* (med. diss., Munich, 1950)

[102] Foucault, *Order of Things* (n. 87), p. 36

[b103-80.4stein] 103.I have given the socioeconomic discourses and practices more thought and attention in my recent monograph (n. 2). Based on that research I am inclined to think that, due to the flexibility of early modern disease definition, generalizations as to the identity of the pox are virtually impossible. More microhistorical studies than this one, I submit, would show that disease identity in the early modern period was always distinctively local. What the pox was—that is, how it was perceived, talked about, and distinguished from other diseases—ultimately depended on the specific sociocultural environment, the various terrains of discourse and practice surrounding disease phenomena within the specific local setting in which they occurred.

